# Supplementary material for: Skin layer-specific spatiotemporal assessment of micrometabolism during wound angiogenesis
Source: Commun Biol. 2024 Dec 20;7:1574. doi: 10.1038/s42003-024-07257-4 (PMC11662067; doi:10.1038/s42003-024-07257-4)
Supplement: Supplementary file 1 — Supplementary Information [file 42003_2024_7257_MOESM1_ESM.pdf]

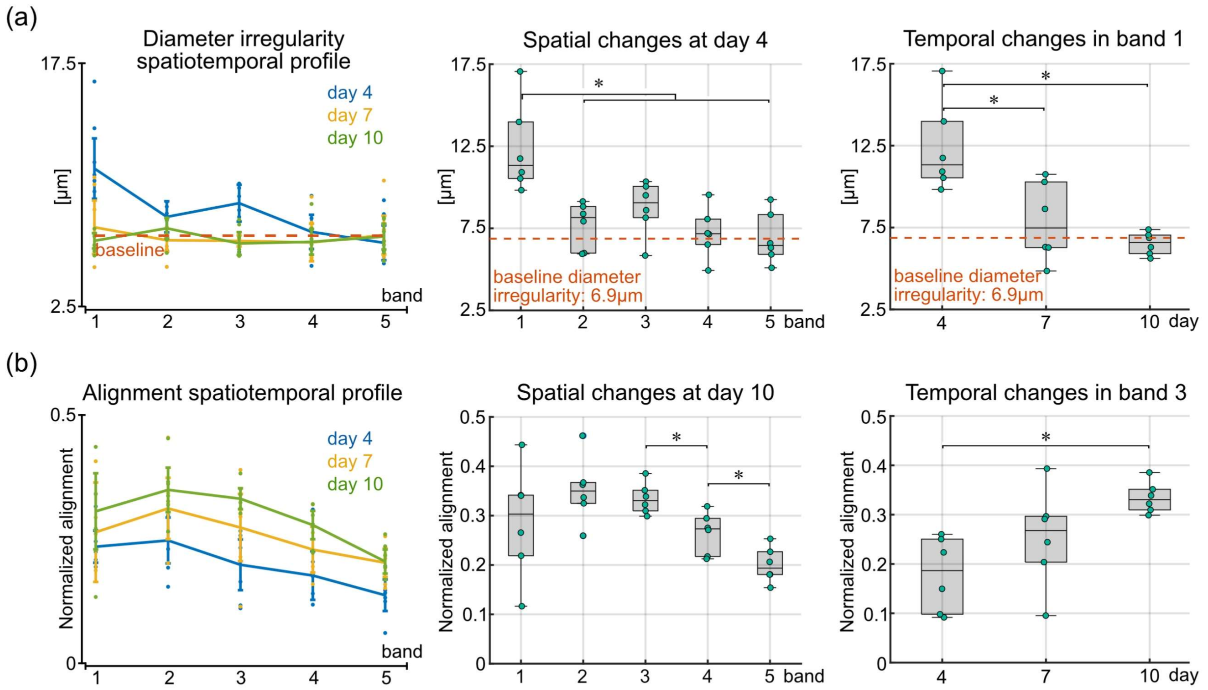

Supplementary Fig. 1: Quantitative analysis of the spatiotemporal profile of vessel diameter irregularity and angular alignment. (a) Spatiotemporal profile of changes in vessel diameter irregularity, defined as the standard deviation of individual diameter values within a region. Line plot on the left presents an overview of the spatiotemporal trend (n = 6 wounds from 4 mice). Baseline measurement was taken on healthy skin of the same group of mice. Middle box plot shows the spatial changes at day 4. Right box plot shows the temporal changes in band 1. (b) Spatiotemporal profile of changes in vessel angular alignment towards the wound center. A normalized value of 1 indicates full alignment, and -1 indicates perpendicular orientation. Left line plot presents an overview of the spatiotemporal trend (n = 6 wounds from 4 mice). Since angular alignment is calculated with respect to the wound center, the baseline measurement on healthy skin is not applicable. Middle box plot shows the spatial changes at day 10. Right box plot shows the temporal changes in band 3. Asterisks (*) indicate significant differences ($p < 0.05$). Error bars in line graphs are defined as 95% confidence intervals.
